# Factors associated with persistent high use of emergency departments: a nationwide retrospective cohort study

**DOI:** 10.1136/bmjopen-2026-123062

**Published:** 2026-10-07

**Authors:** Akshay Kumar, Catriona Marshall, Sonia Saraiva, William Lee, Suzanne Mason, Christopher D Burton, Christina Maria van der Feltz-Cornelis, Robert M West, Chris Bojke, Richard Mattock, Samuel David Relton, Elspeth Guthrie

**Affiliations:** 1 Psychological and Social Medicine, Leeds Institute of Health Sciences, Leeds, UK; 2 College of Medicine and Health, University of Exeter, Exeter, UK; 3 School of Health and Related Research, University of Sheffield, Sheffield, UK; 4 Academic Unit of Primary Medical Care, University of Sheffield, Sheffield, UK; 5 Dept of Health Sciences, YBRI, University of York, York, UK; 6 Hull York Medical School, University of York, York, UK; 7 Academic Unit of Health Economics, University of Leeds, Leeds, UK; 8 HEOR, Lumanity, London, UK; 9 Leeds Institute of Health Sciences, Leeds, UK

**Keywords:** Emergency Departments, STATISTICS & RESEARCH METHODS, MENTAL HEALTH

## Abstract

**Abstract:**

**Objectives:**

To identify patient and attendance characteristics that predict persistent high use of emergency departments (EDs) in England and to quantify the relationship between these factors and the duration an individual remains a high user.

**Design:**

Nationwide retrospective cohort study using a Cox proportional hazards model with random effects.

**Setting:**

EDs across England, using linked national healthcare datasets including hospital admissions, General Practice (GP) prescribing data and mortality records from January 2016 to December 2019.

**Participants:**

The total national dataset comprised approximately 150 million ED attendances by 30 million adult patients (aged>18). From this, a random sample of 5 million individuals was analysed. In the 2017 calendar year, 1 766 208 individuals attended the ED, of which 91 034 (5.2%) met the criteria for high use (defined as five or more attendances within a 12-month period).

**Primary and secondary outcome measures:**

The primary outcome was the length of time an individual remained in the ‘high user’ status. Predictors were measured using HRs to determine the likelihood of transitioning out of high-user status (where an HR<1.00 indicates a longer duration of high use).

**Results:**

Several factors significantly predicted prolonged high-user status. Strong predictors (expressed as HRs) included: use of hypnotics and anxiolytics (HR=0.64), antipsychotics (HR=0.67), opioid analgesics (HR=0.73) and non-opioid analgesics (HR=0.74), being a ‘burst’ attender (HR=0.55), social deprivation (HR=0.81), leaving the department after refusing treatment (HR=0.77), leaving before being treated (HR=0.86) and having ‘nothing abnormal detected’ during the visit (HR=0.88).

**Conclusions:**

Persistent high ED use is strongly associated with prescriptions for analgesics and mental health medications, as well as specific attendance behaviours like ‘burst’ patterns and leaving without treatment. These findings suggest that healthcare providers should prioritise proactive care plans, enhanced mental health support and specialised pain management strategies to intervene before patients become long-term frequent users. Further research into the efficacy of these specific interventions for ‘burst’ attenders is warranted.

**STRENGTHS AND LIMITATIONS OF THIS STUDY**

- To our knowledge, this is the first study which has examined high use of the emergency department (ED) that has analysed the time an individual spends as a high user using time-to-event analyses.
- We used national data involving emergency care and hospital admissions in England over a 3-year period.
- We included GP prescription data about the use of psychotropic medication and analgesia in the 90 days before each ED attendance in our analyses.
- We included ‘burst attendances’ (a sequence of two or more attendances with less than 14 days) between consecutive visits as a dependent variable in our analyses.
- We were not able to explore physical comorbidities as a predictor of persistent high use.

## Background

 People who are high users of the emergency department (ED) present a significant challenge to health services.^1–3^ In England, approximately 9% of adults attending an ED in a year have at least three attendances,^4 5^ 2.5% at least 5 attendances and 0.3% ten or more.^5^ Overall, people attending three or more times account for over a quarter of all attendances in a year.^4^ Similar patterns occur globally,^6 7^ although observed values vary between locations and times.^6^ While most of those who attend multiple times in 1 year do not continue at this level in the next year; a minority continue to be high users over multiple years.^2 8 9^ This persistent high use has received considerably less attention than high use over a single year.

The proportion of high users in 1 year who remain high users in the next varies between studies. Six published studies,^8–13^ and unpublished data from the Yorkshire and Humber region of England (Burton, personal communication), using thresholds of between 4 and 10 attendances, found persistence rates of 19% to 38% with one exception of 53% in a single region of the USA which used a very high threshold (10+attendances).^13^ High users of the ED include people with complex medical conditions, mental health problems, substance-related disorders and complex social problems,^14–16^ occurring both in isolation and in combination. Mental disorders appear to be particularly common in high ED users^9^ and are associated with further consultation in high users.^17^

While studies of intervention for high ED use have typically focused on those with high use during a 1-year period, the high rates of spontaneous resolution mean that interventions may spuriously appear effective.^8^ Focusing on those individuals with, or at elevated risk of, persistent attendance should result in greater attention to those with greatest need and also increase the statistical power of studies that evaluate interventions. For this to be possible, we need to better understand the factors that contribute to persistent high ED use as a prelude to developing targeted interventions aimed at improving patient outcomes and reducing ED visits. Statistical modelling offers a robust approach to identify and analyse the predictors of persistent high ED users. In particular, time-to-event analysis permits the estimation of time spent as a high user as a continuous measure rather than simply representing data as binary states.

We aimed to use routinely collected ED attendance data from hospitals across England to provide new insights into persistent frequent attendance. Our objectives were

1. To describe and visualise patterns of frequent attendance over a 3-year period.
2. To identify factors associated with persistent high ED use employing time-to-event analysis.

## Methods

### Study design

The Frequent Users of Emergency Departments (FUsED) study (project reference 132852) is a large multi-modal study of high intensity ED use and interventions to address it. In this paper we report on a retrospective cohort study using routine administrative data.

### Data sources

This study analysed ED attendances from the National Health Service (NHS) England Accident & Emergency (A&E) Hospital Episode Statistics (HES) dataset^18^ and the Emergency Care Dataset (ECDS) for the period 01 January 2016 to 31 December 2019. ED patterns of attendance were severely disrupted during the calendar year of 2020 because of the COVID-19 pandemic, so we chose not to include data from this period. Linked hospital admissions data from the HES Admitted Patient Care dataset,^19^ mortality statistics from the Office for National Statistics^20^ dataset and primary care prescribing data from the English Prescribing Dataset^21^ were also used in this study. HES is a curated data set which includes data from all hospitals in England and the English Prescribing Dataset contains detailed information on all prescriptions issued in England.

### Patient and public involvement

Patient and public involvement (PPI) forms a pivotal component of the FUsED project with regular PPI meetings conducted to both steer research objectives and disseminate research findings. Patients were involved in the design and conduct of this research. The findings of this study were disseminated to targeted stakeholders from NHS services and to the PPI reference group of the research programme during December 2024 and January 2025.

### Data management

#### Data extraction

The HES dataset received from NHS England contained approximately 30 million individuals and 150 million attendances over the study period. Each ED attendance was linked by pseudonymised patient ID and fiscal year with demographic information including age, sex, ethnicity and Index of Multiple Deprivation (IMD).^22^ The English IMD is the official measure of relative deprivation for small areas in England and ranks every small area in England according to its level of deprivation. Deprivation quintiles are calculated by ranking all the small areas from most deprived (IMD=1) to least deprived (IMD=5). A random sample of 5 million people was extracted for the subsequent pre-processing and analysis. Linked data included GP prescriptions (using the English Prescribing Dataset) and the patient’s date of death was extracted (using the Office for National Statistics Mortality dataset).

#### Reasons for attendance

Primary reasons for attendance were extracted from the HES, A&E and ECDS datasets. To enable consistent analysis, we mapped all primary diagnoses to a unified set of broad attendance reason categories. We first mapped Systematized Nomenclature of Medicine - Clinical Terms (SNOMED CT) codes to HES A&E codes and then condensed both HES A&E and International Statistical Classification of Diseases and Related Health Problems, 10th revision codes to a common diagnostic framework. Full details of the mapping process are provided in supplemental material (online supplemental table S1).

#### Prescriptions

We used GP prescription data to determine whether patients had been prescribed medication in the 90 days before each attendance. It was not possible to examine all prescription data as the dataset would have been too large to analyse. We focused instead on prescriptions for psychotropic medication and analgesics as there is evidence to suggest that high users have high rates of mental health problems and chronic painful conditions.^23^ We used category sections of the British National Formulary (BNF)^24^ to identify analgesic and psychotropic medications. Analgesic medications included opioid analgesics (BNF 4.7.2) and non-opioid analgesics and compound preparations (BNF 4.7.1). Psychotropic drugs included hypnotics and anxiolytics (BNF 4.1), antipsychotics (BNF 4.2) and antidepressant drugs (BNF 4.3). The dictionary of medicines and devices (dm+d) codes were converted to BNF sub-sub-chapters using the National Health Service Business Services Authority mapping file (August 2024 version). The analysis was conducted at the level of individual ED attendances. Medication exposure was therefore defined for each attendance as whether the individual had received a relevant prescription within the previous 90 days. Consequently, medication status could vary between attendances for individuals with repeated ED attendances. To account for the correlation between repeated attendances contributed by the same individual, a patient-level random effect was included in the Cox mixed-effects model.

#### High user status definition

We used a definition for high user of 5 or more attendances within a 12-month period.^16^ We allocated two attendance states: current high user and non-high user. Current high user (CHU) at a threshold of 5 attendances (CHU5) was applied at any attendance which was the fifth or greater attendance in the previous 365 days including the current day. Non-high user indicated that there were less than five attendances in the previous 365-day period. CHU5 status was deemed to persist until the date at which they had less than five attendances in the previous 365-day period. In addition to CHU5 state, we also used a higher threshold of ten or more attendances within 365 days (CH10), which reflects clinical thresholds for referral to high user services in England. For patients defined as high users (either CHU5 or CHU10), the date at which the individual left high user status and time spent in CHU status was derived.

#### Burst attender status definition

In addition to considering the number of attendances, regardless of their timing, we also included the presence of bursts as an indicator of clustering of an individual’s attendances in time.^7^ An ED attendance was said to belong to a burst of attendances if it formed a sequence of two or more attendances with less than 14 days between consecutive visits.^7^ At each ED attendance, a patient was provided a burst attendance status (true) if the above definition held, ‘false’ otherwise. We also derived an ‘ever burst attender’ variable to determine if the patient was defined as a burst attender at any point during the study period.

### Statistical analysis

#### High user cohort characteristics

To characterise and visualise persistent high use, we first isolated patients attending an ED within the period 01 January 2017 and 31 December 2017 (year 1), who were CHU5, and examined their attendance patterns over the next two calendar years (01 January 2018 to 31 December 2018-year 2, and 01 January 2019 to 31 December 2019-year 3). Characteristics of individuals belonging to this cohort were summarised and include sex, age, ethnicity and IMD status (taken at an individual’s first attendance). Descriptive statistics were used to compare sub-groups. We have included p values in the results although, due to the large sample sizes, any difference between groups produces small p values which may detract from assessing the actual size and clinical relevance of group differences.^25^ Alluvial plots were produced to describe future attendance patterns of high users in year 1. Age was categorised using age groups informed by findings from a previous study as part of the FUsED project. Deprivation was categorised into quintiles, consistent with previous studies and the commonly used approach to representing socioeconomic deprivation in UK research. These categorisations were specified a priori for the analysis.

#### Time-to-event analysis

A Cox proportional hazards model with random effects was used to quantify the relationship between patient/attendance characteristics and the length of time an individual was defined as a high user. Time in high user status was censored if the individual died before the date at which high user status ended, or if this date occurred after the study period ended (31 December 2019). Covariates included in the multivariable Cox model were selected based on clinical expertise and findings from previous literature. The selection was made a priori to ensure that variables considered clinically relevant to persistent high ED use were accounted for in the analysis. The patient-level fixed effects considered in this model were age, sex, IMD status, ethnicity and whether the individual had been prescribed analgesics or psychotropic drugs within 90 days prior to the attendance. Attendance-level fixed effects included in this model were the arrival mode, discharge status, burst attendance status and primary diagnosis of the ED attendance. If an attendance was not made between the hours of 08:00 and 18:00 on a working day (Monday to Friday excluding bank holidays) the attendance was said to be made out-of-hours and was also included as a fixed effect in the model. An individual’s patient ID was included as a random effect to account for multiple attendances made by a given individual. Interactions between covariates were considered but not included due to the computational complexity of the multivariable mixed-effects model. The potential for clinically relevant effect modification remains an area for future research. The HRs, corresponding p values, Harrell’s C-Indices and variance explained by the patient-level random effects are presented in this manuscript. The Cox proportional hazards model was fit using the coxme package in R (V.4.4.1). In our analysis, Kaplan-Meier plots represent the probability that an individual remains in high user status at a given time t and is therefore a measure of persistent high use. Out of the randomly selected sample of 5 million individuals over the 3-year study period, there were 129 713 attendances made by patients who attended five or more times within 365 days (CHU5) and 96 926 attendances made by patients who attended 10 or more times (CHU10); it is these attendances included in this time-to-event analysis. The main time-to-event analysis was conducted using the complete case per variable with missing values excluded, and an additional sensitivity analysis using a missing category in the model was also reported. Schoenfeld residuals were plotted per variable to test the assumption of the Cox regression models (online supplemental figures S1 and S2).

## Results

### High user cohort characteristics

Out of the sample of 5 million individuals randomly selected from the original dataset from NHS England, 1 766 208 patients attended ED during the 12-month baseline period (calendar year of 2017). Of this baseline group, 91 034 (5.2%) were defined as a CHU5, and 12 367 (0.7%) as a CHU10. Table 1 summarises the patient characteristics of this baseline group. High users were more likely to live in areas of higher social deprivation than non-high users and this was most apparent for those people attending 10 or more times per year (CHU10) (non-high user 21.1% vs CHU10 28% for the most deprived IMD category). High users were also more likely than non-high users to be prescribed psychotropic medication (CHU5=52.2% vs non-high user=33.3%) and analgesic prescriptions (CHU5=49.9% vs non-high user=30.8%) within 90 days prior to an attendance.

**Table 1:** Patient characteristics of individuals with an ED attendance in 2017: non-high users versus individuals who attended 5 or more times (CHU5) and 10 or more times (CHU10) in a 365-day period

	Non-high users (n=1 662 807)		CHU5 (n=91 034)		CHU10 (n=12 367)		Non-high user versus CHU5 p value	Non-high user versus CHU10 p value
	N	%	N	%	N	%		
Sex								
Male	795 614	47.8	41 883	46	6091	49.3	<0.001	0.002
Female	860 281	51.7	48 795	53.6	6237	50.4	<0.001	0.004
Missing	6912	0.4	356	0.4	39	0.3	0.260	0.084
Age category								
18–29	394 705	23.7	21 077	23.2	2978	24.1	<0.001	0.372
30–64	846 098	50.9	37 645	41.4	5900	47.7	<0.001	<0.001
65+	421 975	25.4	32 311	35.5	3489	28.2	<0.001	<0.001
Missing	29	0.01	1	0.01	–	–	0.647	0.642
Ethnicity								
White	951 322	57.2	70 013	76.9	10 031	81.1	<0.001	<0.001
Asian	74 909	4.5	5678	6.2	709	5.7	<0.001	<0.001
Black	39 584	2.4	3567	3.9	489	4.0	<0.001	<0.001
Mixed	12 803	0.8	1036	1.1	143	1.2	<0.001	<0.001
Other ethnicities	39 651	2.4	2382	2.6	290	2.3	<0.001	0.773
Missing	544 538	32.7	8358	9.2	705	5.7	<0.001	<0.001
IMD quintile								
5=least deprived	294 601	17.7	13 648	15.0	1447	11.7	<0.001	<0.001
4	309 363	18.6	15 663	17.2	1789	14.5	<0.001	<0.001
3	324 376	19.5	17 680	19.4	2393	19.3	0.522	0.659
2	338 194	20.3	20 229	22.2	2898	23.4	<0.001	<0.001
1=most deprived	350 612	21.1	22 540	24.8	3502	28.3	<0.001	<0.001
Missing	45 661	2.7	1274	1.4	338	2.7	<0.001	0.930
Burst attender in period								
Yes	181 392	10.9	56 638	62.2	10 463	84.6	<0.001	<0.001
No	1 481 415	89.1	34 396	37.8	1904	15.4	<0.001	<0.001
Prescribed psychotropic drugs in period								
Yes	554 379	33.3	47 535	52.2	7646	61.8	<0.001	<0.001
No	1 108 428	66.7	43 499	47.8	4721	38.2	<0.001	<0.001
Prescribed analgesic drugs in period								
Yes	512 808	30.8	45 425	49.9	7035	56.9	<0.001	<0.001
No	1 149 999	69.2	45 609	50.1	5332	43.1	<0.001	<0.001

CHU, current high user; ED, emergency department; IMD, Index of Multiple Deprivation.

Table 2 summarises attendance characteristics made by high users and non-high users in the total study period (01 January 2017 to 31 December 2019). There were 8 719 076 attendances during this period, of which 711 431 attendances were made by individuals defined as a CHU5 and 295 859 by individuals defined as a CHU10. The mean time in high user status was 291 days for those defined as CHU5 and 484 days for those defined as CHU10. Table 2 shows that high users were more likely than non-high users to be conveyed to hospital by ambulance (41.4% vs 24.0%). There were relatively few marked differences between groups in terms of primary reason for attendance, except that high users were less likely to make injury-related attendances (CHU5, 16.2% vs non-high users, 25.6%). However, the proportion of high users compared with non-high users prescribed psychotropic medication and analgesic medication was much higher for all types of these drugs: hypnotics and anxiolytics (25.9% vs 12.6%); antipsychotics (14.6% vs 5.2%); antidepressants (50.6% vs 31.8%); non-opioid analgesics (47.6% vs 28.7%); and opioid medication (36.8% vs 19.1%). The differences between very high users (CHU10) and non-high users were even more apparent for all the above categories. There were minimal differences between high users and non-high users in terms of types of discharge except for one category, admission to hospital. CHU5 individuals were more likely to be admitted to hospital than non-high users (30.1% vs 19.6%) but very high users (CHU10) had lower rates of hospital admission (24.1%) than CHU5 individuals. This suggests that CHU10 individuals may have less severe urgent physical health problems than CHU5 individuals.

**Table 2:** Emergency department attendance characteristics for years 01 January 2017 to 31 December 2019: non-high users versus individuals who attended 5 or more times (CHU5) and 10 or more times (CHU10) in a 365-day period

	Non-high user (n=7 711 786)		CHU5 (n=711 431)		CHU10 (n=295 859)		Non-high user versus CHU5 p value	Non-high user versus CHU10 p value
	N	%	N	%	N	%		
Arrival mode								
Ambulance	1 849 338	24.0	294 536	41.4	140 197	47.4	<0.001	<0.001
Other	5 508 603	71.4	386 585	54.3	142 068	48.0	<0.001	<0.001
Missing	353 845	4.6	30 310	4.3	13 594	4.6	<0.001	0.871
Attendance part of burst								
True	1 441 531	18.7	332 221	46.7	224 923	76.0	<0.001	<0.001
False	6 270 255	81.3	379 210	53.3	70 936	24.0	<0.001	<0.001
Out-of-hours								
True	4 143 594	53.7	409 814	57.6	192 951	65.2	<0.001	<0.001
False	3 568 178	46.3	301 616	42.4	102 908	34.8	<0.001	<0.001
Primary reason for attendance								
Injury	1 971 890	25.6	115 050	16.2	44 339	15.0	<0.001	<0.001
Cardiac and vascular conditions	367 340	4.8	36 857	5.2	12 336	4.2	<0.001	<0.001
Central nervous system conditions	122 564	1.6	16 042	2.3	6797	2.3	<0.001	<0.001
Endocrine diseases	37 456	0.5	7448	1.0	3003	1.0	<0.001	<0.001
Ear, nose and throat conditions	180 886	2.3	12 317	1.7	2533	0.9	<0.001	<0.001
Findings not elsewhere classified	3 245 611	42.1	317 062	44.6	141 624	47.9	<0.001	<0.001
Gastrointestinal conditions	334 966	4.3	37 597	5.3	16 196	5.5	<0.001	<0.001
Infections, infectious diseases	99 038	1.3	10 832	1.5	2418	0.8	<0.001	<0.001
Mental health	115 996	1.5	24 995	3.5	21 361	7.2	<0.001	<0.001
Nothing abnormal detected	118 925	1.5	11 917	1.7	5792	2.0	<0.001	<0.001
Other conditions	233 325	3.0	21 131	3.0	8540	2.9	0.009	<0.001
Respiratory	319 065	4.1	43 226	6.1	13 946	4.7	<0.001	<0.001
Unclassified	332 516	4.3	28 146	4.0	8433	2.9	0.009	<0.001
Urinary conditions	232 113	3.0	28 777	4.0	8515	2.9	<0.001	<0.001
Prescribed drugs within 90 days prior to attendance								
Hypnotics and anxiolytics								
True	970 783	12.6	184 060	25.9	108 902	36.8	<0.001	<0.001
False	6 741 003	87.4	527 371	74.1	186 957	63.2	<0.001	<0.001
Antipsychotics and related drugs								
True	400 857	5.2	103 874	14.6	75 477	25.5	<0.001	<0.001
False	7 310 929	94.8	607 557	85.4	220 382	74.5	<0.001	<0.001
Antidepressants								
True	2 454 310	31.8	359 816	50.6	181 898	61.5	<0.001	<0.001
False	5 257 476	68.2	351 615	49.4	113 961	38.5	<0.001	<0.001
Non-opioid analgesics								
True	2 211 163	28.7	338 718	47.6	153 236	51.8	<0.001	<0.001
False	5 500 623	71.3	372 713	52.4	142 623	48.2	<0.001	<0.001
Opioid analgesics								
True	1 475 894	19.1	261 794	36.8	132 218	44.7	<0.001	<0.001
False	6 235 892	80.9	449 637	63.2	163 641	55.3	<0.001	<0.001
Discharge status								
Discharged-no follow-up	3 476 785	45.1	263 578	37.0	119 002	40.2	<0.001	<0.001
Admitted to hospital bed	1 508 825	19.6	214 261	30.1	71 358	24.1	<0.001	<0.001
Discharged-GP follow-up	883 878	11.5	71 817	10.1	30 579	10.3	<0.001	<0.001
Referred to ED clinic	75 431	1.0	6713	0.9	2840	1.0	**0.005**	0.323
Referred to fracture clinic	146 764	1.9	5416	0.8	1264	0.4	<0.001	<0.001
Referred to outpatient clinic	178 406	2.3	12 593	1.8	3886	1.3	<0.001	<0.001
Transferred to other healthcare provider	130 772	1.7	11 663	1.6	5155	1.7	<0.001	0.054
Referred to other healthcare professional	90 279	1.2	7177	1.0	2896	1.0	<0.001	<0.001
Left department before being treated	127 845	1.7	15 261	2.1	13 755	4.6	<0.001	0.001
Left department having refused treatment	13 411	0.2	1988	0.3	1839	0.6	<0.001	<0.001
Other	461 411	6.0	45 324	6.4	20 242	6.8	<0.001	<0.001
Missing	610 322	7.9	54 878	7.7	22 921	7.7	<0.001	0.001

CHU, current high user; ED, emergency department; GP, General Practice.

Figure 1 is an alluvial plot which shows the movement between high users (CHU5) and non-high user status over the 3-year study period. It displays the attendance status of 91 034 individuals defined as a CHU5 in year 1 (2017) over the next 2-year period. Of this cohort, 32 413 (35.6%) were also defined as a CHU5 in year 2 and 14 641 (16.1%) defined as a CHU5 in all 3 years. The alluvial plot also shows that a small proportion of high users in year 1 lose this status in year 2 but regain high user status in year 3. Figure 2 shows three alluvial plots of the high users over 3 years according to age categories (18–29, 30–64 and 65+years) and suggests that older persistent high users have higher mortality rates than younger patients.

**Figure 1:**
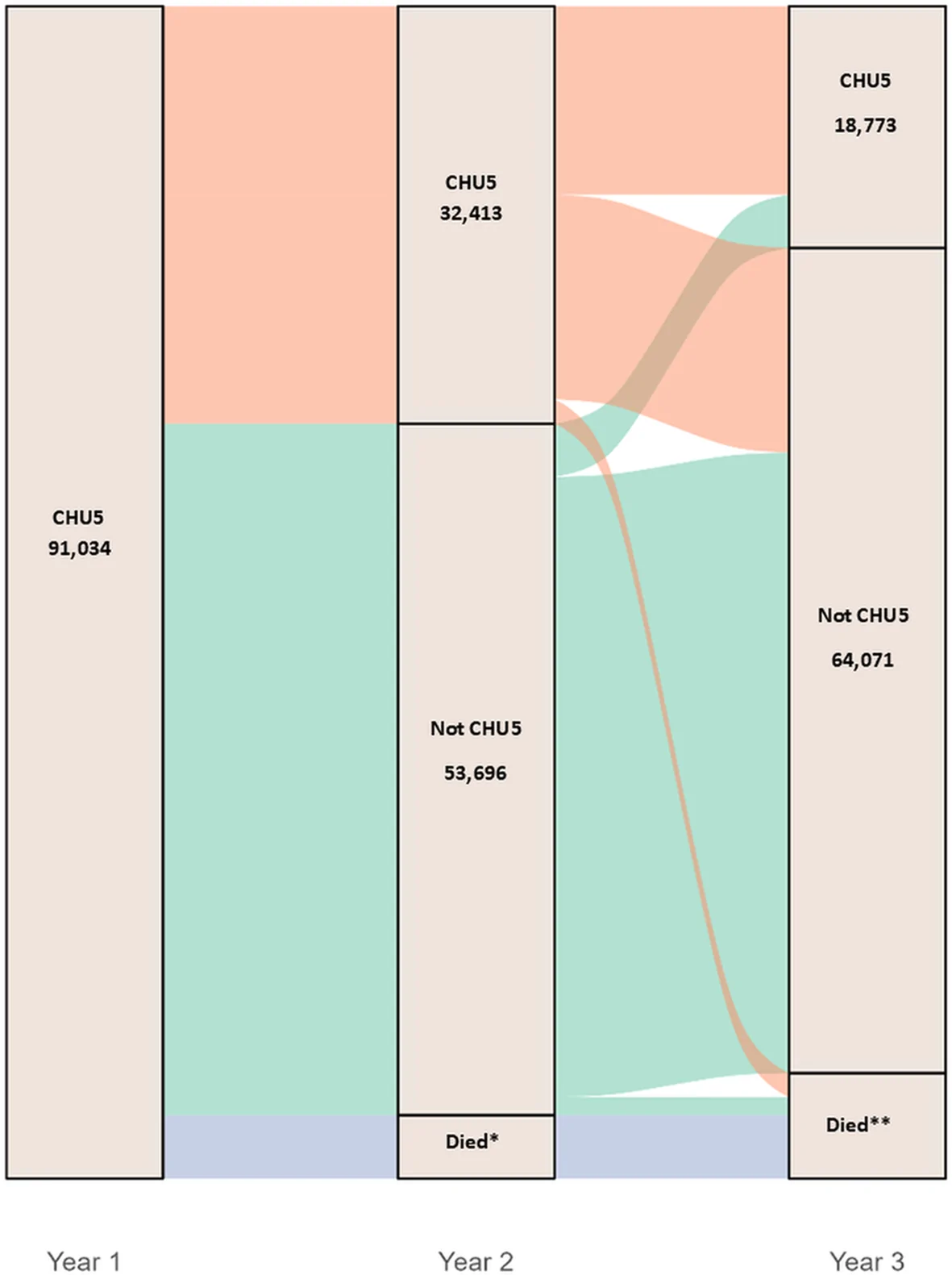
Overall alluvial plot of current high users of emergency departments (with five or more attendances in the previous 365-day period, CHU5) in year 1 (calendar year 2017) and the subsequent CHU status in the following two calendar years (*4925, **8190).

**Figure 2:**
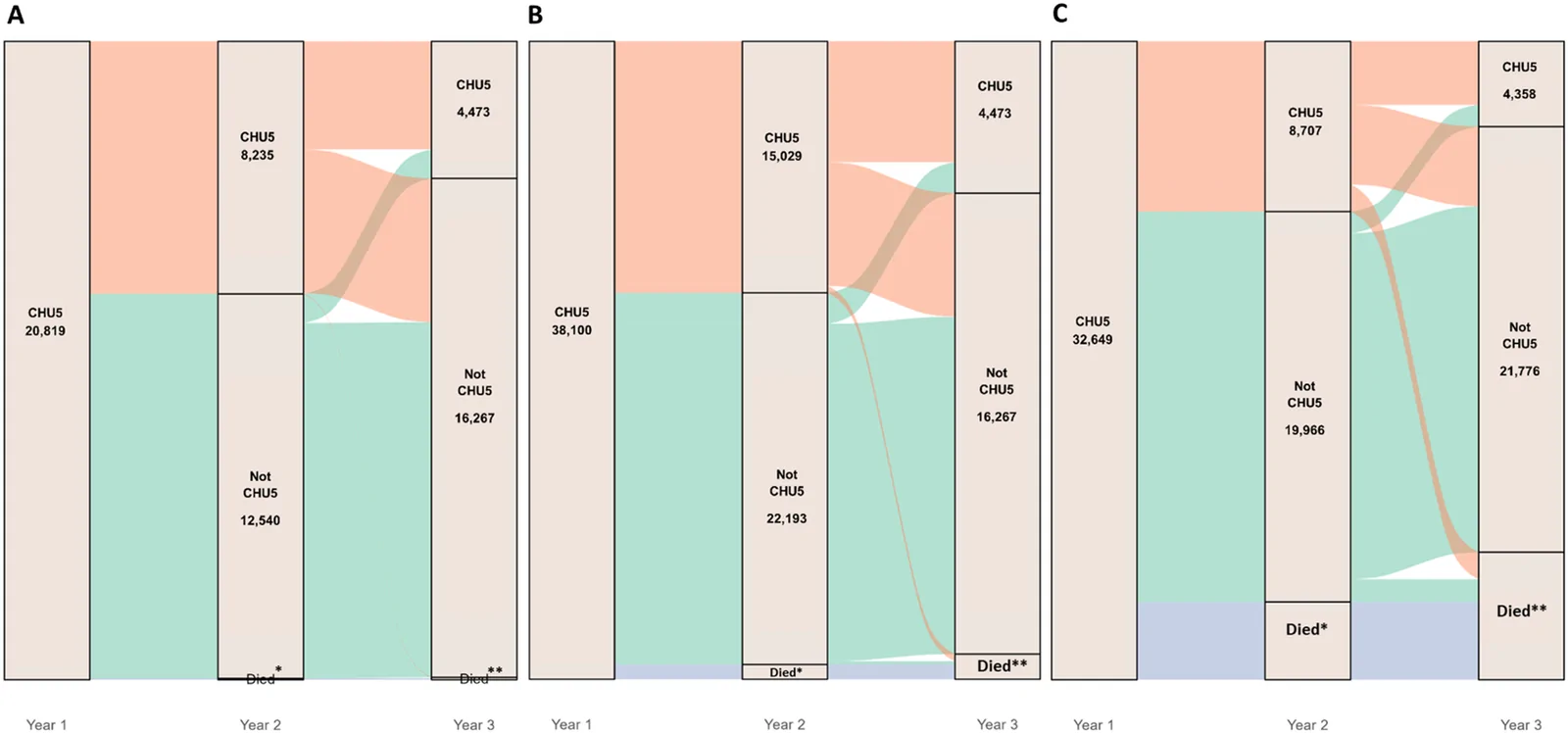
Alluvial plot of current high users of emergency departments (with five or more attendances in the previous 365-day period, CHU5) in year 1 (calendar year 2017) and the subsequent two calendar years by age band in year 1 (A=18–29 years, *=44, **=79, B=30–64 years, *=878, **=1504 and C=65+ years, *=3976, **=6525).

### Time-to-event analysis

Online supplemental table S2 shows the results of the Cox proportional hazards model with random effects. The HRs represent a measure of the fixed effect on the time an individual spent in high user status. Hence, in our case, a HR of less than 1 suggests the fixed effect was associated with a reduced likelihood of high user status ending at a given time t and is therefore a measure of persistent high use. The results from the Cox proportional hazards model with random effects suggest there is considerable variation between patients as the SD reported is 1.69. Strong predictors of persistent high use include being a burst attender (HR=0.55), being prescribed psychotropic or analgesic drugs (with a particularly strong association with being prescribed hypnotics, anxiolytics, non-opioid analgesics and opioid analgesics HR=0.64, 0.67, 0.74 and 0.73, respectively). Greater levels of deprivation (HR=0.81), having nothing abnormal detected during the ED attendance (HR=0.88) and leaving the department having refused treatment was also associated with persistent high use (HR=0.77). However, females and those aged 65+years were less likely to be associated with persistent high use in comparison to males and those aged 18–29 years, respectively (HR=1.27 and HR=1.47, respectively).

Figure 3 shows the Kaplan-Meier estimates of the time until a CHU5 individual leaves high user status for the group as a whole (**A**), burst attenders versus non-burst attenders (**B**), sex (**C**), age bands (**D**), ethnicity (**E**), levels of social deprivation (**F**), psychotropic medication prescriptions (yes/no) (**G**) and analgesic prescriptions (yes/no) (**H**). All these plots display a dip at 365 days, which is due to a combination of attendance patterns and the definition of high use. In particular, there are a number of people who had a short burst of 5+attendances—fulfilling our cohort entry criteria—but then did not visit the ED again for a year and therefore lost their high user status at the 365 day overlap between year 1 and year 2, which causes the dip. The majority of CHU5 individuals lose their high user status within 12 months of first obtaining their status, but the long tail of the distribution indicates a slower reduction in high user status over the next 2 years. Individuals who attended in bursts were much more likely to be high users in year 1 and retain their CHU5 status than non-burst attenders over the next 2 years. The other Kaplan-Meier curves suggest the following groups were more likely to be high users and retain their high user status than their comparator groups: younger and older high users than middle aged; people of white ethnicity than other ethnicities; people with higher levels of social deprivation; people prescribed analgesic medication and people prescribed psychotropic medication.

**Figure 3:**
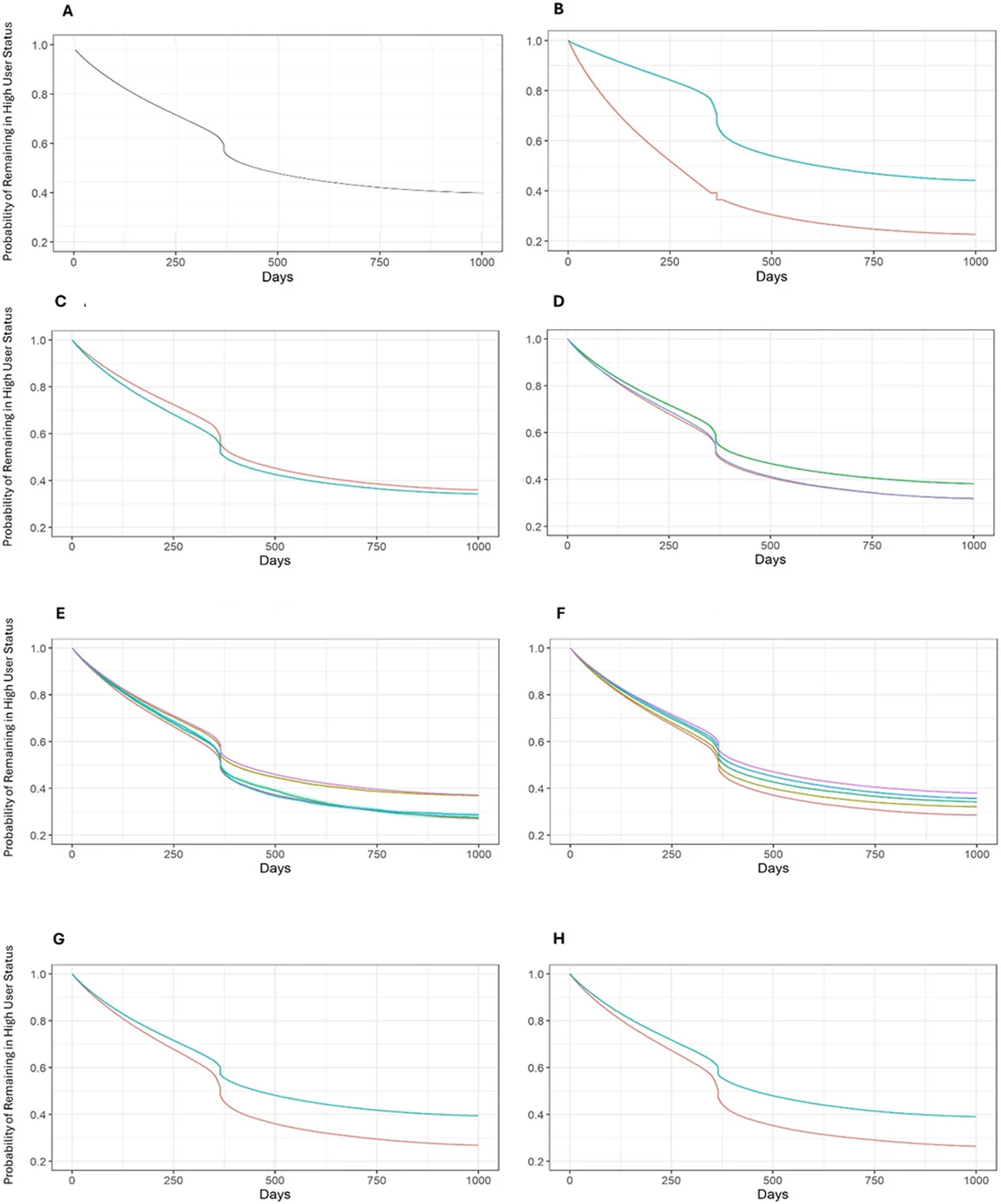
Kaplan-Meier plots showing the probability of an individual remaining in current high user status (attending five or more times in the previous 365 period, CHU5). (A) Overall. (B) Burst attenders (blue=true, red=false). (C) Sex (blue=male, red=female). (D) Age bands (red=19–29, green=30–64, blue=65+). (E) Ethnicity(red=Asian, yellow=black, green=mixed ethnicity, purple=white, blue=other ethnicity). (F) Deprivation status (red=5-least deprived, yellow=4, green=3, blue=2, purple=1 most deprived). (G) Those prescribed psychotropic drugs (blue=true, red=false). (H) Those prescribed analgesics (blue=true, red=false).

### Sensitivity analysis

In general, when considering factors associated with persistent high use using the CHU10 definition instead of CHU5, the differences between the groups and their comparators were more marked for those classified under the 10+definition. For example, being prescribed psychotropic drugs or analgesics had a greater association with persistent high use using the 10+definition in comparison to the 5+definition. However, the pattern was not as clear regarding social deprivation, where there was a reduced association between deprivation and persistent high CHU10 use. The findings of the time-to-event analysis also suggest there may be an association with younger patients and persistent attendance when using 10+definition. The sensitivity analysis conducted using a missing category rather than the complete case data largely showed analogous findings (online supplemental table S3). Notably however, missing sex data were associated with higher risk of persistent high use for both CHU5 and CHU10 definitions. Conversely, missing IMD data were associated with lower risk of persistent high use for both CHU5 and CHU10 definitions.

## Discussion

### Summary of principal findings

This nationwide study explores persistent high use over a 3-year period before the COVID-19 pandemic. Over a third of high users in the calendar year 2017 were also defined as high users in 2018. Those making ED attendances within a short period of time (burst attenders) and those residing in the most deprived areas were strongly associated with both high use and persistent high use. Analgesics and psychotropic drugs prescribed in a primary care setting were stronger predictors of persistent high use than the primary reason for attendance. ‘Nothing abnormal being detected’ in the ED and having left the ED either after refusing treatment or before treatment could be given were predictors of persistent high use.

### Comparison with other studies

The proportion of high users who became persistent high users in this study is consistent with previous studies^9 26 27^ and suggests that over 60% of high users are temporary high users whose attendance will stop without any intervention necessary to reduce ED attendance. Mental health problems,^9 26–29^ chronic pain^26 27^ and social deprivation^9 26 28^ have all been linked previously to persistent high use, as has leaving the ED prematurely.^26 30^

Patterns of attendance have been studied less frequently. An Australian study of linked data from three hospitals found temporal clustering of attendances was more frequent in high users with mental health and alcohol and drugs problems compared with those without but did not examine clustering itself as a risk factor for persistent use.^30^ In the present study, a pattern of burst attendances in the first year of presentation was one of the strongest predictors of persistent use, as were prescribed psychotropic and analgesic drugs. Diagnoses and reasons for attendance at ED are not always accurately recorded, whereas prescription drugs can be proxy indicators of mental health and chronic pain problems. Previous research has established a link between chronic pain, unexplained physical symptoms, and frequent ED use.^31^ In our study, this association seems to be highlighted through the use of linked GP analgesic prescriptions. Given that individuals with chronic conditions and non-communicable diseases are frequently prescribed analgesics for pain management, it is plausible that a substantial proportion of high users are seeking care either directly for pain-related issues or indirectly for the broader management of these underlying conditions.

Mortality has been previously linked with high use of the ED, with high users having a median 2.2-fold increased odds of mortality compared with non-frequent users.^32^ In this study, mortality was clearly associated with older age (65+years) and was not prevalent in young persistent users. Older people nearing the end of life are an especially vulnerable group. Repeated visits to the ED in the last few months of life, often involving long waits in overcrowded departments, are not conducive to high-quality care. Interventions to support complex and refractory symptoms in the frail elderly living at home or in a care home need to be further developed.^33^

We found that men were more likely than women to be persistent high users of ED. However, most studies which examine persistent use report that women are at higher risk of persistent high ED use than men.^26–28 34 35^ One other study has reported that males are at higher risk.^9^ Differences in countries, healthcare systems and study sites are most likely to account for these differences.

### Implications for practice and further research

The study has found some easily identifiable features of individuals who are likely to be persistent attenders. Using this information could lead to strategies that prevent persistent high use through early identification of those at most risk and better targeted care for these individuals in primary and community care settings. It is common for patients who are high users of ED to have several of the risk factors we identified. The risk ratios in the modelling can be combined to obtain an estimate of the likelihood of persistent attendance for a particular individual. This data could be potentially used in clinical practice to help inform local decision making about referral to high user services, although clinical decisions should always be based on the detailed assessment of the high user and their needs at the time.

High use of the ED is a ubiquitous problem in Western Healthcare Services.^36^ Although individual factors influencing this phenomenon may vary according to country and type of healthcare service, there are several findings from this study that are relevant to most clinical settings. These include: the self-limiting nature of high ED use for the majority of high users; the difference in age groups of high users and the association of older high users with increased mortality; and the association of persistent high use with the likelihood of mental health/chronic physical pain factors and burst attendance.

Future research should focus on early identification of individuals through primary care and community care, and the design and testing of interventions that would lead to better personalised management, outcomes and reduced resource use.

### Strengths and limitations

To our knowledge this is the first study that has analysed the time an individual spends as a high user using time-to-event analyses on a nationwide scale. The availability of linked data across three consecutive calendar years also enabled us to visualise patterns of attendances made by high users. Previous studies have outlined that a notable proportion of users have complex healthcare needs and often suffer from the effects of comorbidities. Due to a combination of poor consistency of primary diagnosis coding across hospitals and fiscal years, and the reduced availability of GP records, exploring comorbidities as a predictor of persistent high use was not possible. This study does however provide a method of using medication prescribed in a primary care setting-thus future studies may replicate this approach to derive insights into conditions for those prescribed certain medication. It was planned to explore interactions between variables in the time-to-event model; however, due to the complexity of the model and scale of the dataset, this was not possible at this time. The proportional hazards assumption was broadly satisfied for most covariates; however, some variables, including burst attendance and arrival mode, showed evidence of variation in their effects over time. Although the overall model was retained given its complexity and the generally consistent proportional hazards assumption across covariates, the estimated HRs for these variables should be interpreted with some caution as they represent an average effect over the follow-up period. Future studies could investigate these time-varying effects using models incorporating time-dependent coefficients or alternative approaches to account for non-proportional hazards. Death was treated as a censoring event in the Cox regression analysis. As death precludes subsequent occurrence of the study outcome, competing risks may have influenced the estimated associations, and this should be considered when interpreting the findings. Future studies could consider competing-risk approaches to further investigate this. The COVID-19 pandemic significantly altered and disrupted ED attendances for a period of months in 2020 which meant we had to limit our data analysis to a pre-pandemic period. Following the COVID-19 pandemic, ED attendances returned to previous levels and are now showing signs of increasing year on year.^37 38^ It is highly likely that our findings from the pre-pandemic period are still of relevance

## Conclusion

The findings of this study indicate analgesics prescriptions, mental health related prescriptions and burst attendance are strong predictors of persistent high ED use, which may help healthcare providers implement preventive strategies, such as proactive care plans, mental health support or pain management alternatives, before these patients become long-term high users of healthcare services.

## Supplementary material

10.1136/bmjopen-2026-123062 online supplemental file 1

## Data Availability

Data may be obtained from a third party and are not publicly available.
