# Supplementary material for: Factors associated with persistent high use of emergency departments: a nationwide retrospective cohort study
Source: BMJ Open. 2026 Oct 7;16(10):e123062. doi: 10.1136/bmjopen-2026-123062 (PMC13647028; doi:10.1136/bmjopen-2026-123062)
Supplement: Supplementary file 1 [file bmjopen-2026-123062.reviewer_comments.pdf]

PEER REVIEW HISTORY

BMJ Open publishes all reviews undertaken for accepted manuscripts. Reviewers are asked to complete a checklist review form and are provided with free text boxes to elaborate on their assessment. These free text comments are reproduced below.

ARTICLE DETAILS

Title (Provisional)

Factors associated with persistent high use of emergency departments: A nationwide retrospective cohort study

Authors

Kumar, Akshay; Marshall, Catriona; Saraiva, Sonia; Lee, William; Mason, Suzanne; Burton, Christopher D; van der Feltz-Cornelis, Christina Maria; West, Robert M; Bojke, Chris; Mattock, Richard; Relton, Samuel David; Guthrie, Elspeth

---

VERSION 1 - REVIEW

**Reviewer**	**1**
**Name**	**Ozgur, Emrah**
**Affiliation**	**Marmara Universitesi Tip Fakultesi, Department of Biostatistics**
**Date**	**06-Jul-2026**
**COI**	**none**

Although the study is based on a large nationwide dataset and investigates an important clinical question, several fundamental methodological shortcomings were identified in the statistical analyses. These issues are not limited to reporting deficiencies but involve key aspects of the analytical approach, which may influence the validity of the reported results and conclusions. Therefore, substantial revision is necessary before the manuscript can be considered for publication. My detailed comments are provided below.

1.The proportional hazards assumption of the Cox model was not assessed or reported. Since this is a fundamental assumption of Cox regression, it should be formally evaluated (e.g., using Schoenfeld residuals). If the assumption is violated, alternative modelling strategies, such as stratified Cox regression or models with time-varying effects, should be considered.

2.Approximately one-third of the ethnicity data are missing, yet the manuscript does not explain how these missing values were handled or justify treating them as a separate category. The missing data strategy should be clearly described, and the use of multiple imputation or sensitivity analyses should be considered.

3.Several predictors, particularly medication prescriptions within the previous 90 days, may vary over time. However, it is unclear whether these variables were treated as baseline covariates or as time-dependent covariates. This should be clarified because it directly affects the interpretation of the estimated hazard ratios.

4. Although the rationale for not reporting p-values because of the very large sample size is understandable, reporting the p-values would provide a more complete statistical description of the comparisons and allow readers to better evaluate the observed differences between groups.

5.The manuscript does not describe the strategy used for selecting variables included in the multivariable Cox model. It is unclear whether predictor selection was based on prior clinical knowledge, previous literature, or a predefined modelling strategy.

6.The performance of the fitted Cox model was not reported. Providing an appropriate measure of model discrimination, such as Harrell's C-index, would help readers evaluate the predictive ability of the model.

7.Age and deprivation index were categorised before analysis without providing a justification for the selected cut-points. Since categorisation may reduce statistical power and obscure non-linear relationships, the rationale for this approach should be explained.

8. Potentially important interaction effects were not evaluated. Clinically plausible interactions, such as age × medication use or age × burst attendance, may influence persistent high ED use and should be explored or discussed as a study limitation.

9. Patients who died during follow-up were treated as censored observations. Since death precludes the occurrence of the study outcome, the authors should justify this approach and discuss the potential impact of competing risks on the study findings.

VERSION 1 - AUTHOR RESPONSE

Although the study is based on a large nationwide dataset and investigates an important clinical question, several fundamental methodological shortcomings were identified in the statistical analyses. These issues are not limited to reporting deficiencies but involve key aspects of the analytical approach, which may influence the validity of the reported results and conclusions. Therefore, substantial revision is necessary before the manuscript can be considered for publication. My detailed comments are provided below.

The authors thank the reviewer for this careful and constructive assessment. We appreciate the concerns raised regarding the methodological aspects of the statistical analyses. We have carefully considered each of the points raised and have now made the necessary revisions to strengthen the statistical methodology and improve the clarity and robustness of the manuscript. Detailed responses to each comment are provided below.

1.The proportional hazards assumption of the Cox model was not assessed or reported. Since this is a fundamental assumption of Cox regression, it should be formally evaluated

(e.g., using Schoenfeld residuals). If the assumption is violated, alternative modelling strategies, such as stratified Cox regression or models with time-varying effects, should be considered.

We thank the reviewer for highlighting the importance of assessing the proportional hazards assumption. We have now included the Schoenfeld residual-based diagnostics in the manuscript. The assumption appeared broadly reasonable for the majority of covariates, although some variation over time was observed for burst attendance and arrival mode. Given the overall complexity of the mixed-effects model and the generally consistent findings across the remaining covariates, we retained the prespecified model. We have now reported the assessment and acknowledged the potential for time-varying effects as a limitation, noting that future analyses could consider models with time-varying coefficients. These plots are now included in the appendix (see appendix figures A and B) with the following added to the strengths and limitations:

"The proportional hazards assumption was broadly satisfied for most covariates; however, some variables, including burst attendance and arrival mode, showed evidence of variation in their effects over time. Although the overall model was retained given its complexity and the generally consistent proportional hazards assumption across covariates, the estimated hazard ratios for these variables should be interpreted with some caution as they represent an average effect over the follow-up period. Future studies could investigate these time-varying effects using models incorporating time-dependent coefficients or alternative approaches to account for non-proportional hazards."

2.Approximately one-third of the ethnicity data are missing, yet the manuscript does not explain how these missing values were handled or justify treating them as a separate category. The missing data strategy should be clearly described, and the use of multiple imputation or sensitivity analyses should be considered.

The authors would like to thank you for this comment and have now clarified the handling of missing data in the revised manuscript. The primary analysis was conducted using complete-case data. As a sensitivity analysis, missing data was instead included as a separate category within the model. The results were analogous between the two approaches, indicating that the findings were not materially affected by the handling of missing ethnicity data, however the missing categories were associated with persistent high use risk and this has been added to the text:

"The sensitivity analysis conducted using a missing category rather than the complete case data largely showed analogous findings (see appendix Table B). Notably however, missing sex data was associated with higher risk of persistent high use for both CHU5 and CHU10 definitions. Conversely, missing IMD data was associated with lower risk of persistent high use for both CHU5 and CHU10 definitions."

3.Several predictors, particularly medication prescriptions within the previous 90 days, may vary over time. However, it is unclear whether these variables were treated as baseline covariates or as time-dependent covariates. This should be clarified because it directly affects the interpretation of the estimated hazard ratios.

We thank the reviewer for highlighting this point. The analysis was conducted at the level of individual ED attendances, rather than at the patient level. Medication use was defined as a binary attendance-level variable, indicating whether the individual had received a relevant

prescription within the 90 days preceding that specific ED attendance. Therefore, medication exposure could vary between attendances for individuals with repeated ED attendances. A patient-level random effect was included in the Cox mixed-effects model to account for the correlation between repeated attendances contributed by the same individual. We have clarified this in the revised manuscript.

"The analysis was conducted at the level of individual ED attendances. Medication exposure was therefore defined for each attendance as whether the individual had received a relevant prescription within the previous 90 days. Consequently, medication status could vary between attendances for individuals with repeated ED attendances. To account for the correlation between repeated attendances contributed by the same individual, a patient-level random effect was included in the Cox mixed-effects model."

4. Although the rationale for not reporting p-values because of the very large sample size is understandable, reporting the p-values would provide a more complete statistical description of the comparisons and allow readers to better evaluate the observed differences between groups.
We thank the reviewer for this helpful suggestion. We have now included the p-values in the revised table to provide a more complete statistical description of the comparisons (See table 1 and table 2).

5.The manuscript does not describe the strategy used for selecting variables included in the multivariable Cox model. It is unclear whether predictor selection was based on prior clinical knowledge, previous literature, or a predefined modelling strategy.

We thank the reviewer for highlighting this point. Variable selection for the multivariable Cox model was based on clinical knowledge and findings from previous literature. We have now clarified this in the revised manuscript.

"Covariates included in the multivariable Cox model were selected based on clinical expertise and findings from previous literature. The selection was made a priori to ensure that variables considered clinically relevant to persistent high ED use were accounted for in the analysis."

6.The performance of the fitted Cox model was not reported. Providing an appropriate measure of model discrimination, such as Harrell's C-index, would help readers evaluate the predictive ability of the model.

We thank the reviewer for this helpful suggestion. Harrell's C-index has now been included in the revised tables to provide a measure of the discrimination of the fitted Cox model (See Table 3 and appendix Table B).

7.Age and deprivation index were categorised before analysis without providing a justification for the selected cut-points. Since categorisation may reduce statistical power and obscure non-linear relationships, the rationale for this approach should be explained.

We thank the reviewer for highlighting the rationale for categorising age and deprivation. Age categories were based on findings from a previous study, while deprivation was categorised into

quintiles, consistent with previous studies and the commonly used approach to representing socioeconomic deprivation in UK research. We have now clarified the rationale for these categorisations in the revised manuscript.

"Age was categorised using age groups informed by findings from a previous study (39). Deprivation was categorised into quintiles, consistent with previous studies and the commonly used approach to representing socioeconomic deprivation in UK research. These categorisations were specified a priori for the analysis."

8. Potentially important interaction effects were not evaluated. Clinically plausible interactions, such as age × medication use or age × burst attendance, may influence persistent high ED use and should be explored or discussed as a study limitation.

We thank the reviewer for highlighting the potential importance of interaction effects. Clinically plausible interactions, including age × medication use and age × burst attendance, were considered but were not included due to the computational complexity of the multivariable mixed-effects model. The potential for clinically relevant effect modification therefore remains an area for future research. This is included as a limitation:

"It was planned to explore interactions between variables in the time-to-event model, however due to the complexity of the model and scale of the dataset this was not possible at this time"

9. Patients who died during follow-up were treated as censored observations. Since death precludes the occurrence of the study outcome, the authors should justify this approach and discuss the potential impact of competing risks on the study findings.

We thank the reviewer for highlighting the potential competing risk of death. Death was treated as a censoring event in the primary Cox regression analysis, as individuals who died could no longer experience the study outcome. We have now clarified this approach in the revised manuscript and acknowledged the potential impact of competing risks on the interpretation of the findings as a limitation.

"Death was treated as a censoring event in the Cox regression analysis. As death precludes subsequent occurrence of the study outcome, competing risks may have influenced the estimated associations, and this should be considered when interpreting the findings. Future studies could consider competing-risk approaches to further investigate this."

VERSION 2 - REVIEW

**Reviewer**	**1**
**Name**	**Ozgur, Emrah**
**Affiliation**	**Marmara Universitesi Tip Fakultesi, Department of Biostatistics**
**Date**	**15-Sep-2026**
**COI**	

I would like to thank the authors for their careful and thoughtful revision of the manuscript. The biostatistical comments I raised in my previous review have been adequately addressed, and the revisions have improved the methodological clarity and statistical reporting of the study. I have no further biostatistical concerns and wish the authors success with their work.
