# Supplementary material for: Factors associated with persistent high use of emergency departments: a nationwide retrospective cohort study
Source: BMJ Open. 2026 Oct 7;16(10):e123062. doi: 10.1136/bmjopen-2026-123062 (PMC13647028; doi:10.1136/bmjopen-2026-123062)
Supplement: Supplementary file 2 [file bmjopen-16-10-s001.docx]

**Appendix**

##### **Table S1**-**Diagnosis category groupings**

| **Reason for Attendance** | **ICD-10 Codes** | **ICD-10 Category** | **HES Codes** | **HES Category** |
| --- | --- | --- | --- | --- |
| Injury | **S codes**  **T codes**  **M79.9** | Injury, poisoning and certain other consequences of external causes  Soft tissue disorder, unspecified  Injury non-mental health) | **01-16** | Laceration, Contusion/abrasion, Soft tissue inflammation, Head injury, Dislocation/fracture/joint injury/amputation, Sprain/ligament injury, Muscle/tendon injury, Nerve injury, Vascular injury, Burns and scalds, Electric shock, Foreign body, Bites/stings, Poisoning (inc overdose), Near drowning, Visceral injury |
| Respiratory Conditions | **J codes** | Diseases of the respiratory system | **25** | Respiratory conditions |
| Infections and infectious diseases | **A codes**  **B codes**  **L08.9** | Certain infectious and parasitic diseases  Local infection of skin and subcutaneous tissue, unspecified | **17**  **18**  **19** | Infectious disease  Local infection  septicaemia |
| Gastrointestinal Conditions | **K codes**  **R10.0** | Diseases of the digestive system  Abdominal and pelvic pain | **26** | Gastrointestinal conditions |
| ENT Conditions | **H codes** | Diseases of the eye and adnexa  Diseases of the ear and mastoid process | **34** | ENT conditions |
| Central Nervous System conditions | **G codes** | Diseases of the nervous system | **24** | Central nervous system conditions (exc stroke) |
| Urinary Conditions | **N30-N39** | Symptoms and signs involving the urinary system | **27** | Urinary conditions |
| Cardiac and Vascular conditions | **I codes** | Diseases of the circulatory system | **20**  **21**  **22**  **23** | Cerebro-vascular conditions  Other vascular conditions  Haematological conditions |
| Mental Health | **F codes**  **R45**  **X71 – X83** | Mental and behavioural disorders and self harm | **35**  **37** | Psychiatric  Social problems |
| Endocrine conditions | **E codes**  **E10-E14** | Diabetes mellitus | **301**  **302** | Diabetes  Non-diabetic |
| Other conditions | **R50-R69**  **All other codes left after above** | General symptoms and signs | **31**  **32**  **33**  **36** | Dermatological  Allergy (inc anaphylaxis)  Faciomaxillary conditions  Ophthalmological |
| Findings not elsewhere classified | **‘none’** |  | **38 or ‘none’** |  |
| Nothing abnormal detected |  |  | **39** |  |
| Unclassified | missing |  | missing |  |

**Table S2- Time to Event Analysis, Cox Proportional Hazards Model (hazard ratio, HR <1 suggests the fixed effect was associated with a reduced likelihood of current high user status, CHU ending at a given time t and is therefore a measure of persistent high use)**

|  | CHU5  (N=129,713) | | CHU10 (N=96,926) | |
| --- | --- | --- | --- | --- |
|  | HR | 95% CI | HR | p-value |
| Sex (Ref=Male) |  |  |  |  |
| Female | 1.27 | (1.21, 1.33) | 1.45 | (1.36, 1.54) |
|  |  |  |  |  |
| Age Category (Ref=18-29) |  |  |  |  |
| 30-64 | 1.25 | (1.20, 1.31) | 1.56 | (1.46, 1.66) |
| 65+ | 1.45 | (1.36, 1.54) | 2.68 | (2.48, 2.89) |
|  |  |  |  |  |
| Ethnicity (Ref=White) |  |  |  |  |
| Asian | 1.11 | (1.05, 1.17) | 1.09 | (1.01, 1.18) |
| Black | 1.00 | (0.97, 1.03) | 0.81 | (0.64, 1.02) |
| Mixed | 1.03 | (0.94, 1.12) | 0.86 | (0.71, 1.04) |
| Other Ethnicities | 0.95 | (0.86, 1.05) | 1.03 | (0.99, 1.07) |
|  |  |  |  |  |
| IMD Quintile (Ref=5, Least Deprived) |  |  |  |  |
| 4 | 0.96 | (0.92, 1.00) | 1.02 | (0.98, 1.06) |
| 3 | 0.85 | (0.81, 0.89) | 1.05 | (0.99, 1.11) |
| 2 | 0.85 | (0.81, 0.89) | 1.07 | (1.01, 1.13) |
| 1=Most Deprived | 0.81 | (0.77, 0.85) | 1.06 | (1.01, 1.12) |
|  |  |  |  |  |
| Burst Attendance (Ref=No) | 0.55 | (0.52, 0.59) | 0.58 | (0.52, 0.63) |
|  |  |  |  |  |
| Patient Prescribed Drugs in Previous 90 Days |  |  |  |  |
| Hypnotics and Anxiolytics (Ref=No) | 0.64 | (0.60, 0.68) | 0.61 | (0.57, 0.65) |
| Antipsychotics (Ref=No) | 0.67 | (0.63, 0.71) | 0.67 | (0.63, 0.71) |
| Antidepressants (Ref=No) | 0.88 | (0.84, 0.92) | 0.85 | (0.81, 0.89) |
| Non-opioid Analgesics (Ref=No) | 0.74 | (0.70, 0.78) | 0.66 | (0.62, 0.70) |
| Opioid Analgesics (Ref=No) | 0.73 | (0.69, 0.77) | 0.68 | (0.64, 0.72) |
|  |  |  |  |  |
| Attendance discharge status (Ref=No follow-up) |  |  |  |  |
| Admitted to hospital bed | 0.89 | (0.85, 0.93) | 0.91 | (0.87, 0.95) |
| Discharge with GP follow-up | 0.82 | (0.78, 0.86) | 0.79 | (0.75, 0.83) |
| Referred to ED clinic | 0.77 | (0.73, 0.81) | 0.77 | (0.73, 0.81) |
| Referred to fracture clinic | 0.84 | (0.80, 0.88) | 0.81 | (0.77, 0.85) |
| Referred to outpatient clinic | 0.82 | (0.78, 0.86) | 0.83 | (0.79, 0.87) |
| Transferred to other healthcare provider | 0.92 | (0.88, 0.96) | 0.94 | (0.86, 1.02) |
| Referred to other healthcare professional | 0.81 | (0.77, 0.85) | 0.77 | (0.73, 0.81) |
| Left department before being treated | 0.86 | (0.82, 0.90) | 0.83 | (0.79, 0.87) |
| Left department having refused treatment | 0.77 | (0.73, 0.81) | 0.77 | (0.73, 0.81) |
| Other | 1.05 | (1.02, 1.08) | 1.08 | (1.04, 1.12) |
|  |  |  |  |  |
| Attendance arrival mode (Ref=Non-ambulance) |  |  |  |  |
| Ambulance (including air ambulance) | 0.96 | (0.92, 0.99) | 0.99 | (0.96, 1.02) |
| Not known | 1.28 | (1.22, 1.34) | 1.29 | (1.23, 1.35) |
|  |  |  |  |  |
| Attendance out-of-hours (Ref=No) | 0.99 | (0.97, 1.01) | 0.98 | (0.97, 0.99) |
|  |  |  |  |  |
| Primary Reason for Attendance (Ref=Injury) |  |  |  |  |
| Cardiac/vascular Conditions | 0.98 | (0.94, 1.02) | 0.99 | (0.96, 1.02) |
| Central Nervous System Conditions | 1 | (0.97, 1.03) | 0.97 | (0.93, 1.01) |
| Endocrine Conditions | 0.93 | (0.87, 0.99) | 1.12 | (1.05, 1.19) |
| Ear Nose & Throat Conditions | 1 | (0.97, 1.03) | 1.07 | (1.00, 1.14) |
| Gastrointestinal Conditions | 0.94 | (0.91, 0.97) | 0.99 | (0.97, 1.01) |
| Infections/Infectious Disease | 1.08 | (1.02, 1.14) | 1.10 | (1.02, 1.18) |
| Mental Health | 0.96 | (0.93, 0.98) | 0.98 | (0.94, 1.02) |
| Respiratory Conditions | 1.01 | (0.98, 1.04) | 1.05 | (1.01, 1.09) |
| Urinary Conditions | 0.99 | (0.96, 1.02) | 1.07 | (1.03, 1.11) |
| Nothing Abnormal Detected | 0.88 | (0.84, 0.92) | 0.90 | (0.86, 0.94) |
| Other Conditions | 0.91 | (0.87, 0.95) | 0.95 | (0.92, 0.98) |
| Unclassified | 0.96 | (0.93, 0.99) | 0.99 | (0.96, 1.02) |
| Findings not Elsewhere Classified | 0.95 | (0.91, 0.99) | 0.99 | (0.96, 1.02) |
|  |  |  |  |  |
| Patient Standard Deviation | 1.69 |  | 1.58 |  |
| Concordance | 0.87 |  | 0.84 |  |

**Table S3-** **Time to Event Analysis, Cox Proportional Hazards Model (hazard ratio, HR <1 suggests the fixed effect was associated with a reduced likelihood of current high user status, CHU ending at a given time t and is therefore a measure of persistent high use) sensitivity analysis with missing category included**

|  | CHU5  (N=343,658) | | CHU10 (N=267,958) | |
| --- | --- | --- | --- | --- |
|  | HR | 95% CI | HR | 95% CI |
| Sex (Ref=Male) |  |  |  |  |
| Female | 1.28 | (1.24, 1.32) | 1.44 | (1.38, 1.50) |
| Missing | 0.42 | (0.33, 0.52) | 0.67 | (0.47, 0.96) |
|  |  |  |  |  |
| Age Category (Ref=18-29) |  |  |  |  |
| 30-64 | 1.21 | (1.17, 1.26) | 1.46 | (1.40, 1.52) |
| 65+ | 1.39 | (1.33, 1.44) | 2.46 | (2.33, 2.60) |
| Missing | 1.07 | (0.35, 3.28) | 5.39 | (1.28, 22.66) |
|  |  |  |  |  |
| Ethnicity (Ref=White) |  |  |  |  |
| Asian | 1.08 | (1.01, 1.14) | 1.14 | (1.06, 1.23) |
| Black | 0.98 | (0.91, 1.06) | 0.81 | (0.71, 0.93) |
| Mixed | 0.96 | (0.83, 1.09) | 1.08 | (0.98, 1.19) |
| Other Ethnicities | 0.90 | (0.83, 1.05) | 0.95 | (0.81, 1.19) |
| Missing | 1.14 | (1.08, 1.20) | 1.08 | (0.84, 1.08) |
|  |  |  |  |  |
| IMD Quintile (Ref=1, Most Deprived) |  |  |  |  |
| 2 | 1.02 | (0.99, 1.05) | 0.98 | (0.95, 1.00) |
| 3 | 1.02 | (0.99, 1.06) | 0.96 | (0.92, 0.99) |
| 4 | 1.12 | (1.08, 1.16) | 0.95 | (0.91, 0.99) |
| 5=Least Deprived | 1.16 | (1.11, 1.20) | 0.95 | (0.91, 1.00) |
| Missing | 1.57 | (1.53, 3.28) | 1.48 | (1.44, 1.53) |
|  |  |  |  |  |
| Burst Attendance (Ref=No) | 0.52 | (0.51, 0.53) | 0.54 | (0.53, 0.55) |
|  |  |  |  |  |
| Patient Prescribed Drugs in Previous 90 Days |  |  |  |  |
| Hypnotics and Anxiolytics (Ref=No) | 0.65 | (0.62, 0.66) | 0.66 | (0.63, 0.68) |
| Antipsychotics (Ref=No) | 0.68 | (0.65, 0.71) | 0.71 | (0.68, 0.75) |
| Antidepressants (Ref=No) | 0.88 | (0.85, 0.90) | 0.91 | (0.88, 0.95) |
| Non-opioid Analgesics (Ref=No) | 0.76 | (0.74, 0.78) | 0.66 | (0.64, 0.68) |
| Opioid Analgesics (Ref=No) | 0.72 | (0.69, 0.74) | 0.71 | (0.69, 0.74) |
|  |  |  |  |  |
| Attendance discharge status (Ref=Referred to Healthcare Professional) 11 |  |  |  |  |
| Admitted to hospital bed 1 | 1.11 | (1.05, 1.16) | 1.16 | (1.10, 1.23) |
| Discharge with GP follow-up 2 | 1.02 | (0.98, 1.08) | 1.02 | (0.96, 1.07) |
| Discharge with no follow-up3 | 1.22 | (1.16, 1.30) | 1.26 | (1.19,1.32) |
| Referred to ED clinic 4 | 0.97 | (0.90, 1.04) | 1.38 | (0.91, 1.07) |
| Referred to fracture clinic 5 | 1.05 | (0.97, 1.13) | 1.03 | (0.93, 1.13) |
| Referred to outpatient clinic 6 | 1.04 | (0.98, 1.10) | 1.07 | (1.00, 1.14) |
| Transferred to other healthcare provider 7 | 1.14 | (1.08, 1.21) | 1.21 | (1.13, 1.29) |
| Left department before being treated 12 | 1.05 | (0.99, 1.10) | 1.07 | (1.01, 1.13) |
| Left department having refused treatment 13 | 0.94 | (0.87, 1.03) | 0.98 | (0.91, 1.06) |
| Other 14 | 1.30 | (1.23, 1.36) | 1.38 | (1.31, 1.46) |
| Missing | 1.33 | (1.26, 1.40) | 1.38 | (1.30, 1.46) |
|  |  |  |  |  |
| Attendance arrival mode (Ref=Non-ambulance) |  |  |  |  |
| Ambulance (including air ambulance) | 0.96 | (0.95, 0.98) | 0.99 | (0.98, 1.09) |
| Not known | 1.11 | (1.08, 1.14) | 1.06 | (1.02, 1.10) |
|  |  |  |  |  |
| Attendance out-of-hours (Ref=No) | 0.99 | (0.98, 1.01) | 0.98 | (0.96, 0.99) |
|  |  |  |  |  |
| Primary Reason for Attendance (Ref=Injury) |  |  |  |  |
| Cardiac/vascular Conditions | 0.99 | (0.96, 1.04) | 0.99 | (0.95, 1.01) |
| Central Nervous System Conditions | 1 | (0.96, 1.04) | 0.97 | (0.94, 1.02) |
| Endocrine Conditions | 0.91 | (0.85, 0.96) | 1.09 | (1.02, 1.15) |
| Ear Nose & Throat Conditions | 1 | (0.96, 1.05) | 1.10 | (1.04, 1.18) |
| Gastrointestinal Conditions | 0.95 | (0.92, 0.98) | 0.99 | (0.96, 1.02) |
| Infections/Infectious Disease | 1.07 | (1.02, 1.12) | 1.10 | (1.03, 1.17) |
| Mental Health | 0.94 | (0.92, 0.97) | 0.97 | (0.94, 0.99) |
| Respiratory Conditions | 1 | (0.98, 1.03) | 1.05 | (1.02, 1.09) |
| Urinary Conditions | 0.98 | (0.96, 1.01) | 1.07 | (1.03, 1.11) |
| Nothing Abnormal Detected | 0.89 | (0.84, 0.92) | 0.90 | (0.86, 0.94) |
| Other Conditions | 0.91 | (0.88, 0.94) | 0.93 | (0.90, 0.97) |
| Unclassified | 0.97 | (0.94, 1.01) | 1.02 | (0.98, 1.06) |
| Findings not Elsewhere Classified | 0.95 | (0.93, 0.96) | 0.99 | (0.97, 1.03) |
|  |  |  |  |  |
| Patient Standard Deviation | 1.63 |  | 1.58 |  |
| Concordance | 0.85 |  | 0.82 |  |

**Figure S1-Schoenfield residual-based plots of the estimated time-varying coefficients, with 95% confidence bands for CFA5 Cox mixed effects model. BNF 4.1=Hypnotics and anxiolytics, BNF 4.2=Antipsychotics and related drugs, BNF 4.7.1=Antidepressants, BNF 4.7.2=Non-opioid analgesics and BNF 4.7.3 =Opioid analgesics, OOH=out-of-hours**

**Figure S2-Schoenfield residual-based plots of the estimated time-varying coefficients, with 95% confidence bands for CFA10 Cox mixed effects model. BNF 4.1=Hypnotics and anxiolytics, BNF 4.2=Antipsychotics and related drugs, BNF 4.7.1=Antidepressants, BNF 4.7.2=Non-opioid analgesics and BNF 4.7.3 =Opioid analgesics, OOH=out-of-hours**
